# HIVsirDB: A Database of HIV Inhibiting siRNAs

**DOI:** 10.1371/journal.pone.0025917

**Published:** 2011-10-11

**Authors:** Atul Tyagi, Firoz Ahmed, Nishant Thakur, Arun Sharma, Gajendra P. S. Raghava, Manoj Kumar

**Affiliations:** Bioinformatics Centre, Institute of Microbial Technology (CSIR), Chandigarh, India; George Mason University, United States of America

## Abstract

**Background:**

Human immunodeficiency virus (HIV) is responsible for millions of deaths every year. The current treatment involves the use of multiple antiretroviral agents that may harm patients due to their toxic nature. RNA interference (RNAi) is a potent candidate for the future treatment of HIV, uses short interfering RNA (siRNA/shRNA) for silencing HIV genes. In this study, attempts have been made to create a database HIVsirDB of siRNAs responsible for silencing HIV genes.

**Descriptions:**

HIVsirDB is a manually curated database of HIV inhibiting siRNAs that provides comprehensive information about each siRNA or shRNA. Information was collected and compiled from literature and public resources. This database contains around 750 siRNAs that includes 75 partially complementary siRNAs differing by one or more bases with the target sites and over 100 escape mutant sequences. HIVsirDB structure contains sixteen fields including siRNA sequence, HIV strain, targeted genome region, efficacy and conservation of target sequences. In order to facilitate user, many tools have been integrated in this database that includes; i) siRNAmap for mapping siRNAs on target sequence, ii) HIVsirblast for BLAST search against database, iii) siRNAalign for aligning siRNAs.

**Conclusion:**

HIVsirDB is a freely accessible database of siRNAs which can silence or degrade HIV genes. It covers 26 types of HIV strains and 28 cell types. This database will be very useful for developing models for predicting efficacy of HIV inhibiting siRNAs. In summary this is a useful resource for researchers working in the field of siRNA based HIV therapy. HIVsirDB database is accessible at http://crdd.osdd.net/raghava/hivsir/.

## Introduction

HIV/AIDS is a major global health threat as 33.3 million peoples around the world were living with HIV by the end of December 2009 and this number is increasing with alarming rate [UNAIDS reports: http://www.unaids.org/en/media/unaids/contentassets/documents/factsheet/2010/20101123_FS_Global_em_en.pdf. In 2009 alone, 2.6 million people were newly infected and 1.8 million AIDS related deaths occurred. AIDS was initially noticed in 1981 and its causative agent HIV was discovered in 1983 [1], [2], [3]. HIV is a positive strand RNA retrovirus which has very high genetic variability due to its fast replication (10^9^ to 10^10^ virions every day) and high mutation rate of ∼3×10^−5^ per nucleotide base per cycle of replication [4]. These mutations resulted in the generation of many different strains of HIV [5], [6].

Currently, there is no drug that completely cures HIV infection (http://www.lanl.gov/discover/curing_aids) and also no vaccine to prevent from future infection [7]. There are few antiretroviral drugs that can slow the disease progression by inhibiting the function of proteins involved at different stages of the HIV life-cycle. However, after span of time HIV become resistant to even these drugs due to high rate of genetic mutation. Moreover, even highly active antiretroviral therapy HAART (a combination of three-four antiretroviral drugs) cannot totally eradicate the viruses as some reside as latent reservoir in the host genome and remain in dormant stage [8].

The continuing threat of HIV infection and non-availability of complete cure necessitate the search for new approaches to curb HIV infection. The recent discovery of RNA interference (RNAi) mechanism in mammalian cells [9] raises the possibility to harness this as a therapeutic tool against HIV [10], [11]. Whenever, cell encounters double stranded RNAs it triggers the RNAi response in which dicer enzyme recognizes and cleaves dsRNAs into duplexes of 19 to 21 nucleotides called siRNA. One strand of siRNA is loaded into multi-protein RNA Induced Silencing Complex (RISC) and act as guide sequence. siRNA-RISC complex recognize the target mRNA or viral RNA having perfect Watson-Crick base pairing and promote ribonuclease mediated degradation of the targeted RNA [12].

The potential of RNAi to inhibit HIV infection was first demonstrated in 2002 by several studies [10], [11], [13]. Subsequently, a number of studies have been carried out using siRNAs and shRNAs targeting HIV genome regions such as tat, rev, gag, pol, nef, vif, env, vpr, and the long terminal repeat (LTR) in infected cells and showed promising results to inhibit viral production [14], [15], [16], [17], [18]. However, it has been shown that knockdown effect (efficacy) of siRNA varies according to its sequence and target site on mRNA and hence results in limited number of highly potent siRNAs. A large number of experimental studies have been carried out in order to understand the features associated with effective siRNAs and these features are implemented to develop algorithms to predict potent siRNAs [19], [20], [21], [22]. There are few databases of siRNAs such as: (a) siRecords [23], (b) siRNAdb [24], (c) HuSiDa [25] that focus on targeting genes of human and other mammals.

In the last decade, numerous studies have reported the use of siRNAs and shRNAs to inhibit the HIV. However, to best of authors' knowledge there is no database of siRNAs/shRNAs targeting HIV. So, it's difficult for the researchers to search and analyze the data from the literature. Therefore, it becomes very important to develop a comprehensive database in order to facilitate research on potential RNAi based therapeutics against HIV. Hence, a manually curetted database, “**HIVsirDB”**, has been developed with information of experimentally validated published siRNAs and shRNAs targeting various HIV genome regions.

## Materials and Methods

### Data source

The database collection is entirely based on data gathered from published siRNA related studies. For the making of the collection of siRNA or shRNA targeting HIV, queries were made on the PubMed database (http://www.ncbi.nlm.nih.gov/sites/entrez?db=PubMed/) with keywords “((siRNA) OR shRNA) AND HIV)”. It retrieves almost 700 articles including 97 reviews as on 14^th^ March, 2011. These articles were examined and unrelated articles (siRNA targeting host gene, reviews and some methodological articles where no siRNA experiments were reported) were eliminated. Articles in non-English languages were also excluded. The remaining articles were carefully scrutinized. Furthermore, position of target in the HIV genome is cross checked with NCBI accession number. If NCBI accession of genome is not given, it was retrieved using information of HIV-strain, position and sequence of target sites. A request was made to the respective authors for efficacy in numerical values or extracted if it's given in graphical format. Thus, data of more than 750 siRNAs/shRNAs targeting HIV were collected.

### Data structure

**Figure 1:**
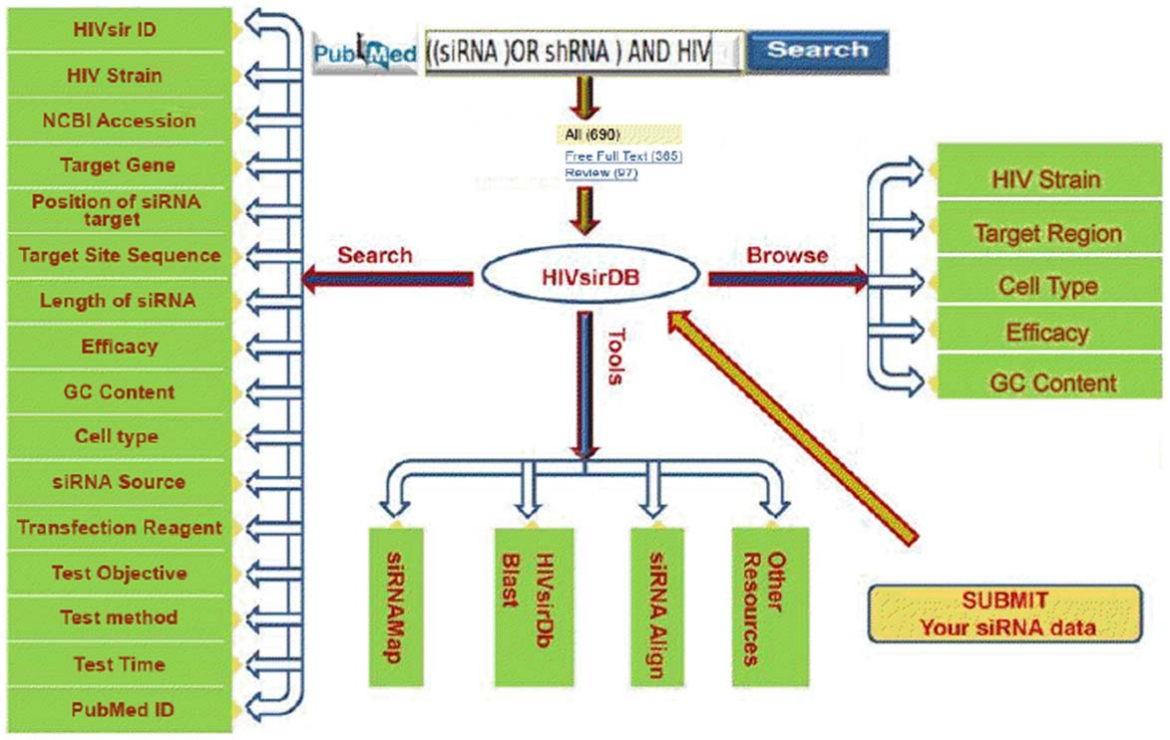
A schematic representation of architecture of database HIVsirDB.

The HIVsirDB contains the following sixteen fields for each siRNAs entry as shown in the **figure 1**; (i) HIVsir ID, (ii) HIV Strain, (iii) NCBI Accession, (iv) Target Gene, (v) Position of siRNA target, (vi) Sense Sequence of siRNA, (vii) Length of siRNA, (viii) Efficacy (%), (ix) GC Content (%), (x) Cell Line, (xi) siRNA Source, (xii) Transfection Reagent, (xiii) Test Objective, (xiv) Test Method, (xv) Test Time (Hours), (xvi) PubMed. Two additional fields have been included in the main database to accommodate information about the conservation of the target sequences and escape studies.

### Web interface and application

The HIVsirDB has been created using open source software LAMP (Linux-Apache-Mysql-PHP) server technology. PHP, HTML and CSS technologies have been used to build the web interface. MySQL, an object-relational database management system (RDBMS), works at the backend and provides commands to retrieve and store siRNAs data into database. PHP a server side scripting language provides interface and functions to fetch and displays data from the database. The whole software system runs on IBM SAS ×3800 machine under Red Hat Enterprise Linux 5 environment using Apache httpd server. PHP and MySQL combination is quite efficient and powerful for database management.

## Results

**Figure 2:**
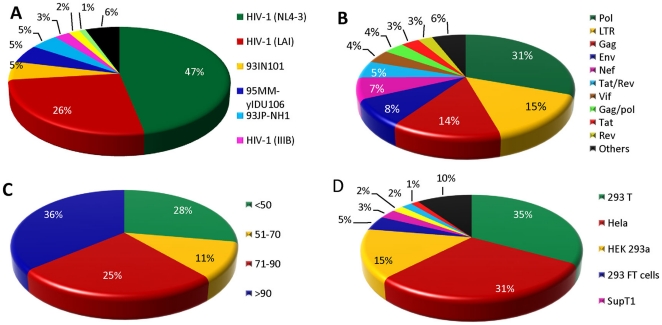
Statistics of important fields included in HIVsirDB. (A) HIV strains. (B) HIV genome regions targeted. (C) Efficacy of siRNA/shRNA. (D) Cell line used.

HIVsirDB database contains manually curated entries of around 750 siRNA/shRNAs against HIV-1 as per literature search till 14^th^ March, 2011. These entries also include 75 partially complementary siRNAs/shRNAs which were differing by one or more bases with the target sites. Database have siRNA entries targeting as many as 26 different HIV stains but majorly HIV-1 NL4-3 and LAI strains were used. Although database has siRNA targeting every regions of HIV genome but predominant regions were Pol, LTR and gag. 293T and Hela cell line were the preferred cell lines used in the experiments. siRNA/shRNA used were of different lengths ranging from 18 to 27 mer, however majority were of 19 or 21 mer. Almost 36% entries of the database constitute of highly efficacious (90–100%) siRNAs. The statistics of the database is given in **figure 2**.

HivsirMut contains information of 107 escape mutants sequences reported for 41 siRNAs available in the main database. It includes details of siRNA and escape target sequence; efficacy of escape mutant; type and number of escape mutation. One, two and three substitutions were reported in 66, 16 and 10 escape sequences respectively (total 88 sequences) and in 14 escape sequences deletions of one or more nucleotide were reported. As many as in 5 sequences, entire 19 bp target region was deleted. In 4 escape sequences both substitutions and deletions were reported. Numbers of escape mutations at each positions of HIV sequence targeted by siRNA are given in **table S1**. Maximum 12 escape substitution mutations were observed at position 8 and 9 while maximum deletion mutations were found at position 5.

HivsirMut also have information of 75 partially complementary siRNAs entries which include 55 single mismatches, 8 double mismatch, 9 triple mismatches and 4 multiple mismatch. In total, there are 111 mismatch or mutation pairs reported. These mismatches were present as different HIV strains were tested for the same siRNA or mutated siRNAs were tested on the same HIV strain. Number of mismatches between siRNA and the HIV target sequence at different positions are given in **table S1**. Maximum of 15 mismatches were observed at position 12.

### Utility Tools

For the better retrieval and analysis of the siRNA data from database we have integrated various tools as search, advance search and browsing. Besides, siRNA mapping and alignment tools were also implemented to further enhance the scope of database. Web tools integrated in HIVsirDB are as follow:

### Keyword search and advance search

A simple text search tool is provided for searching all fields or on selected fields of database and users are free to display all or selected fields. The advance search provides users the best way to search database making various combinations of fields using logical operators “AND & OR” to make advance query for getting more refined results. An export function (CSV) for search results is also included to help the users in sorting the data.

### Browse

This tool helps the user to retrieve siRNA information from database by browsing five important fields viz. HIV Strain, Target Genome Region, Cell Type, Efficacy, and GC Content. This option makes the interface of database more user-friendly.

### siRNA Analysis Tools

**Figure 3:**
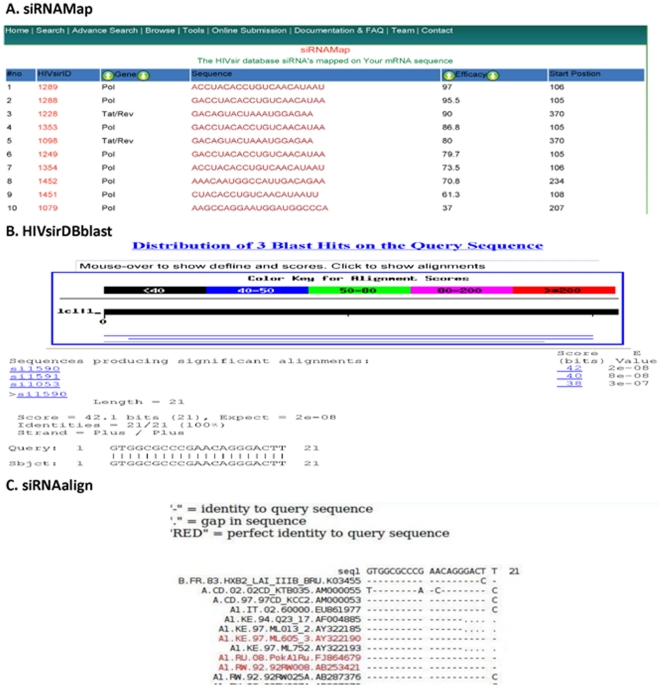
Output of siRNA analysis tools. (A) siRNAmap to map siRNA on user provided HIV sequence. (B) HIVsirDBblast to provide similar siRNA sequences in the database. (C) siRNAalign to find the siRNA sequence variation among different HIV strains.

siRNAmap, HIVsirblast, siRNAalign are three important siRNA analysis tools provided in HIVsirDB. It also has links for the other siRNA resources. In siRNAmap interface user will provide any genome region/gene sequence of HIV and output will show mapping of siRNA/shRNA entries from our database on the user provided sequence. Result shows how many siRNAs have been reported earlier for that sequence along with efficacy and respective positions. Clicking on any result entry, user will get complete information available for that siRNA in the HIVsirDB. HIVsirblast tool search similar siRNAs in HIVsirDB along with pairwise comparison with the user input siRNA sequence. siRNAalign tool align the user provided siRNA sequence with the 1496 HIV reference sequences (available at http://www.hiv.lanl.gov/content/sequence/HIV/mainpage.html) using QuickAlign. Output of siRNAalign in the form of multiple sequence alignment shows the siRNA target sequence conservation or variation among different HIV strains and helps the user to choose the best siRNA. External links of other Non-HIV siRNA database and general siRNAs prediction programs etc. are also provided to help the users. Output of the siRNA analysis tools is shown in figure 3.

### Online submission

In order to add more siRNAs entries specific to HIV genome, the HIVsirDB has online submission option for the users. The user can add new siRNAs/shRNAs related information within specified fields and subsequently entries will be added to the database after proper validation.

## Discussion

RNAi has shown great potential to investigate gene functions and as a potential new class of drugs to suppress disease-causing genes. The beauty of the system that makes it a powerful tool lies in sequence specificity towards particular gene, its quick effect, easiest and most cost effective. RNAi based potential therapeutics would offer advantage over traditional drug molecules as 1) RNAi can target any gene so eventually every protein, but traditional drugs majorly target only certain classes of proteins e.g. G-protein-coupled receptors and ion channels etc. 2) RNAi can block the production of disease-causing proteins before they are made, therefore have greater potential in disease control and intervention. 3) Most importantly, the junction of two protein producing genes as well as regulatory elements could be an important target site, which is only possible by siRNA. 4) As the HIV genome integrates into host genome and persists thus there is great potential to inactivate only HIV genome by using siRNA based approaches.

Unfortunately, HIV may escape the effect of siRNA by mutations in the siRNA target sequences. These escape mutations can decrease or altogether abolish the siRNA inhibition effect. We have collected 107 such HIV escape mutant sequences targeted by 41 siRNAs. Substitution mutations are observed in majority of sequences and clustered around the middle region of the target sequence i.e. 8 to 11 positions. It seems that escape substitution mutations are preferred in the middle region than flanking regions of the target sequences. Simultaneously, deletion mutations were scattered through all the positions in the target sequences.

The mutation generated at target site or nearby results either (A) lack of 100% complementarities between target and siRNA [26], [27] or (B) target site forms a secondary structure and thus not accessible for siRNAs that abolish the siRNA effect [28]. To overcome escape mutants, design of siRNAs targeting highly conserved genome regions that are essential for virus life cycle are preferred. Another promising approach is co-expressing multiple shRNAs that simultaneously target different regions of the viral genome [29], [30], [31] or inhibition of HIV-1 replication with RNAi against cellular co-factors [32].

We have checked the conservation of target sequences of each siRNA among 1496 HIV reference sequences. Analysis showed that for only 1% siRNA were conserved in over 90% target sequences. It could be attributed to high variation among HIV reference sequences. For 13% siRNA target sequences conservation was between 80–90% while 45% siRNA has less than 50% target conservation.

In the database, most of the siRNA efficacy was observed in 293T and Hela cell lines. One potential problem could be the interpretation of these results when comparing them to the natural infection in primary cells. We have analyzed a few siRNA experiments carried out in both cell lines as well as in the primary cells (peripheral blood mononuclear cells: PBMCs [33], [34], peripheral blood lymphocytes: PBLs [10], [35] and CD4+ T cells etc. [36], [37]).

Chang et al. reported the efficient inhibition of HIV infection (>90%) using siRNAs targeting highly conserved pol and vpu sequences against different strains of HIV-1 in 293T cells and marked inhibition effect in primary PBMCs [35]. Sang-Kyung Lee et al. showed that three anti-HIV shRNAs targeting rev, gag, and vif reduced the p24 level by more than 90%, demonstrating their ability to protect primary CD4^+^ T cells, which are the major targets of HIV-1 infection *in vivo*, from homologous virus as they did for HeLa-CD4 cells [37]. Jean-Marc Jacque et al. also described inhibition of early and late steps of HIV-1 replication in Magi cells and primary PBLs by six siRNAs targeted to LTR, vif and nef regions of the HIV-1 genome [10]. These studies suggest that that siRNA perform equally well in both cell lines and primary cells.

RNAi has shown great potential of providing a new class of antiviral therapeutic molecules. Sequence-specific degradation of viral RNA by siRNAs/shRNA gives promising results for the treatment of HIV infection. Various studies have shown selective inhibition of viral genes/proteins that are crucial for HIV-1 replication through transiently expressed synthetic siRNA or continuous expression of vectors containing shRNAs expression cassettes. However, there is no single platform having complete information about siRNA used against HIV. This results in delay in the literature mining, especially in the presence related articles describing siRNA targeting the host genes. So as to speedup research, we have developed HIVsirDB database having comprehensive information about the siRNA/shRNA used in the past targeting every region of the HIV genome.

Users can explore information about the siRNAs/shRNAs sequences, target HIV genome region, efficacies and the experimental conditions prior their experiments in user friendly manner using the search and browsing facility. siRNA analysis tools will help the users to map siRNA on their target sequences and to know the siRNA sequence conservation among 1496 reference strains. Simultaneously, HivsirMut will provide effect of escape mutations and nucleotide mismatch between siRNA and target on the potency. This information would help in picking up the best and effective siRNAs and target genome region for further research.

HIVsirDB, the freely available open source database, would be very useful to experimentalists in deciding the highly potent siRNAs targeting the most susceptible target site in HIV genome and its experimental procedure to suppress HIV infection. The online submission facility will be helpful for updating this database.

### Limitations and future prospects

Our major limitation in developing the database is that the information about siRNAs/shRNAs targeting HIV are too scattered. Extensive literature search is required to further expand the database as numbers of articles were on siRNA/shRNA targeting the host organism.

In future, HIVsirBD would be expanded by including the comprehensive information about siRNAs targeting host genes and their effect on viral entry and propagation. Data of the multiple siRNA targeting different genome regions simultaneously would also be integrated. This database will be updated manually as soon as enough data will be available.

### Availability and requirements

HIVsirDB is available at http://crdd.osdd.net/raghava/hivsir/. To access HIVsirDB World Wide Web is a prerequisite.

## Supporting Information

Table S1 **Number of Escape Mutations/mismatches at different positions in the target sequences.**
(XLS)
